# Swimming Performance and Behavior of High-Altitude Fish in High-Flow Velocity Environments

**DOI:** 10.3390/ani15223327

**Published:** 2025-11-18

**Authors:** Kaixiao Chen, Guanxi Ding, Yun Li, Gangwei He, Yanteng Zhou, Xiaogang Wang

**Affiliations:** 1 Hydraulic Engineering Department, Nanjing Hydraulic Research Institute, Nanjing 210029, China; ckx98@outlook.com (K.C.); yli@nhri.cn (Y.L.); 2 Shaoxing Cao-E River Basin Center, Shaoxing 312366, China; hgw1983@126.com (G.H.); ff888zyt@163.com (Y.Z.)

**Keywords:** *Schizothorax oconnori*, the Tibetan Plateau, high-velocity environment, swimming performance, behavior

## Abstract

The optimization of fishway design relies on a deep understanding of fish swimming performance and behavioral traits. Traditional methods often underestimate fish swimming performance and overlook their behavior under high-flow conditions, particularly in the context of high-altitude species. This study, based on an open-channel flume system and combined with high-speed video tracking and Acoustic Doppler Velocity (ADV) measurements, constructs a Resource Selection Function-Generalized Additive Mixed Models (RSF-GAMMs) to quantify the swimming performance and behavior mechanisms of the high-altitude species, *Schizothorax oconnori* Lloyd, 1908 (*S. oconnori*), in high-velocity environments. The results show that *S. oconnori* significantly outperforms traditional swimming tests and exhibits strong dependence on movement modes. Endurance analysis reveals the breakpoints of endurance models, indicating the species’ high sensitivity to variations in exercise intensity, showcasing the unique physiological and behavioral characteristics of high-altitude fish. In high-velocity conditions, adult *S. oconnori* primarily aims to optimize energy conservation and stability, selectively choosing water bodies with varying disturbance levels depending on its movement mode and endurance state, thus optimizing path selection. This study presents a systematic method for quantifying the extreme swimming abilities and nonlinear behavioral responses of adult *S. oconnori* under complex flow conditions, providing scientific guidance for setting hydraulic thresholds and developing protection strategies for fishways.

## Simple Summary

Fishways are crucial structures that help fish navigate obstacles like dams. Their design largely depends on our understanding of fish swimming performance and behavior. Traditional methods often underestimate fish capabilities, especially neglecting the performance and behavior of high-altitude species in fast-flowing waters. This study focuses on a high-altitude fish species called *Schizothorax oconnori* Lloyd, 1908 (*S. oconnori*) from the Tibetan Plateau. Using flume experiments and behavioral models, the study accurately quantifies the fish’s swimming performance and complex decision-making behaviors in fast currents. The results show that these fish can swim much faster than previously thought, and they cleverly choose their swimming paths based on their movement mode and endurance state to conserve energy and maintain stability. This research provides scientific guidance for setting hydraulic thresholds and developing protection strategies for fishways.

## 1. Introduction

The loss of longitudinal connectivity in rivers and habitat fragmentation have become widespread ecological challenges globally. It is estimated that approximately 48% of rivers worldwide are moderately to severely affected by flow regulation or river segment barriers [1]. Many migratory fish species are unable to reach crucial spawning habitats due to obstacles such as dams and weirs [2,3]. Fishways, as an important measure to restore river connectivity and alleviate migration barriers for fish, have been widely applied around the world [4]. However, the actual effectiveness of fishways often falls short of expectations. One key reason for this is our limited understanding of the swimming performance and behavioral mechanism of target fish species [5].

In the study of fish swimming performance, existing tests often use enclosed swim chambers to measure fish endurance under fixed or progressively increasing single flow conditions [6,7,8]. However, due to limitations in spatial scale and uniform water flow conditions, fish are unable to fully demonstrate their true potential and cannot exhibit natural, energy-efficient swimming strategies such as “burst-and-coast swimming”. As a result, these tests often underestimate the fish’s actual abilities [9]. Studies have shown that, in larger open-channel flumes, which more closely resemble natural environments, fish’s burst swimming speed and endurance significantly improve, with measurements much higher than those obtained in traditional enclosed swim chambers [10]. Additionally, incorporating survival analysis methods into swimming performance assessments allows for the inclusion of individuals who did not exhibit fatigue or reached the endpoint, thus avoiding the underestimation of fish performance by traditional statistical methods, which tend to ignore these “non-fatigued” samples [11,12]. However, it should be noted that commonly used behavioral recording techniques, primarily based on Passive Integrated Transponder (PIT) antenna arrays, have relatively low spatiotemporal resolution, which may lead to conservative estimates of fish capabilities [13]. Using high-definition video tracking for trajectory identification can further improve the accuracy of these estimates.

Fish swimming behavior is profoundly influenced by hydraulic factors such as flow velocity (*V_f_*), turbulent kinetic energy (*TKE*), and Reynolds shear stress (τ). An appropriate flow velocity gradient serves as a clear guide during fish upstream migration [14], while excessively high flow velocity becomes a barrier [15]. To cope with high-flow challenges, fish have evolved the “burst-and-coast swimming” strategy [16], which is 4 to 6 times more energy-efficient than continuous sprinting [17,18,19]. Moderate turbulent kinetic energy can be utilized by fish to reduce movement costs [20], while high levels of turbulent kinetic energy increase swimming costs and negatively impact swimming performance [21]. Reynolds shear stress significantly affects fish swimming performance and stability [22,23], with extremely high levels potentially leading to severe injury or death [24]. The impact of this stress depends on its direction relative to the fish body, with the longitudinal component having the greatest effect [22]. To further quantify fish behavioral preferences for different hydraulic factors, field-based prototype observational experiments combining hydraulic and positional data can provide insights into the conditions fish experience while moving in their environment. Methods such as Step Selection Functions (SSF) [25,26] or Resource Selection Functions (RSFs) [27,28] are commonly used to estimate habitat preferences by statistically comparing the environmental parameters of actual versus potential locations. Due to the limitations of field monitoring, current analyses typically operate at a minute-scale time step [27], while fish in high-velocity environments can often sustain movement for durations measured in seconds [29]. This necessitates sub-second scale analysis, making indoor flume experiments an ideal method for studying fish behavioral preferences in high-velocity environments. Additionally, most existing models based on generalized linear models [25,26,27,28,30] struggle to fully capture the nonlinear threshold effects commonly present in fish behavioral responses [31,32,33]. Therefore, there is an urgent need to develop analytical models with nonlinear effects for fine-scale fish behavior preference analysis.

Fish behavioral decisions are closely related to their current movement mode (upstream movement, cross-stream movement, or downstream movement) and endurance state. During upstream movement, under low to moderate flow conditions, fish typically choose to actively move through local low-velocity areas, resulting in a relatively winding swimming path. However, when in high-velocity areas, individuals no longer spend time searching for low-velocity channels but instead rapidly sprint through them [34,35]. Cross-stream movement is often triggered by complex flow conditions, with fish moving slowly to search for a better upstream path [36]. In downstream movement, fish typically adopt a “flow-assisted” passive strategy, using the current’s force to reduce the need for active swimming, which leads to more stable path selection, significantly shorter transit times, and a considerable reduction in overall energy expenditure [37,38,39]. A fish’s current endurance state directly influences its behavioral decisions—when energetic, fish tend to continue upstream movement or cross turbulent areas to find a better route, whereas as fatigue accumulates, fish are more likely to rest in low-velocity areas or even temporarily retreat downstream to seek rest [40]. Current studies mainly consist of large-scale field surveys [36,41], with conclusions largely qualitative and lacking quantitative results, limiting their direct applicability to fishway design and habitat restoration. Research based on near-natural open-channel flumes allows for detailed measurement of hydraulic conditions and fine tracking of fish behavioral trajectories, deepening the study of how fish behavior is influenced by multiple factors under complex flow conditions.

*S. oconnori*, a cold-water fish species endemic to Tibet, is primarily distributed in the main and tributary streams of the Yarlung Tsangpo River and its affiliated waters [42]. It plays a vital role in local fisheries and food webs, and serves as a key species for studying the adaptation mechanisms of high-altitude fish. The species exhibits typical high-altitude adaptation characteristics: in order to survive and migrate in the high-altitude fast currents and low-oxygen environments, these fish have partially degenerated scales on their bodies, developed skin mucus glands, and possess a thicker subcutaneous fat layer to reduce water resistance, enhance sensory perception, and improve cold resistance [43,44,45]. Against the backdrop of highly engineered rivers, habitat changes, and overfishing, wild populations of *S. oconnori* have shown a significant decline and are listed as a vulnerable species in the “Red Book of Endangered Animals of China” [46]. Despite the significant ecological value and conservation importance of this species, research on its real swimming performance and behavioral ecology in open-channel flumes remains limited [47], especially in terms of detailed data on its movement abilities and behavioral decisions under high-flow conditions.

In response to the above needs, this study focuses on *S. oconnori* to explore its swimming performance and behavioral response mechanisms in complex high-flow conditions of open-channel flumes. The research objectives are: (1) to scientifically quantify the real swimming performance of *S. oconnori*; and (2) to investigate the relationship between the fish’s behavioral decisions in high-flow environments and hydraulic factors, movement modes, and endurance states.

## 2. Materials and Methods

### 2.1. Study Apparatus

**Figure 1:**
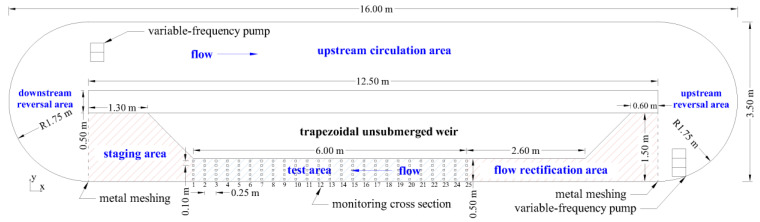
Recirculating open-channel flume (top view), showing the main dimensions of the staging area, test area, flow rectification area, and other circulating areas, with detailed layout dimensions for the flow velocity measurement points (square markers) located at each monitoring cross-section.

The swimming performance and behavioral experiments were conducted in a recirculating open-channel flume located at the Zangmu fish breeding and release station in Tibet, China. The flume is 16.00 m long × 3.50 m wide, and has a 0-degree slope (as shown in Figure 1). It consists of two centrally placed rectangular sections and two semicircular sections. A trapezoidal, non-submersible baffle structure creates a narrow passageway 8.60 m long within the flume, which allows for the shaping of a higher test flow velocity while avoiding turbulence caused by abrupt shape changes. The system is designed with a circulating water flow, where the water passes through the following areas in sequence: a flow rectification area (4.20 m long, 0.50 m to 1.50 m wide), a test area (6.00 m long × 0.50 m wide), a staging area (2.30 m long, 0.50 m to 1.50 m wide), a downstream reversal area (radius 1.75 m), and then enters the upstream circulating area on the northern side (12.50 m long × 1.50 m wide). The water then flows through the upstream reversal area (radius 1.75 m) before returning to the flow rectification area. The entire flume is constructed from smooth, transparent, high-strength acrylic panels (thickness 0.05 m to 0.20 m) to reduce turbulence, flow friction, and boundary layer effects, while also allowing for simultaneous monitoring of fish activity from the side and top. The water flow is powered by variable-frequency pumps (HANTENG, QY70-11-3, Taizhou, China, 0 Hz to 200 Hz) located on both sides of the flume (three pumps on the southeast side and two on the northwest side). The pumps’ power can be adjusted through frequency converters (Clvdrives, C0520H-E, Shenyang, China, 380 V) to precisely control the flow velocity within the flume. The southeast pumps provide the direct power source for the experiment, while the northwest pumps supply adequate power for the upstream circulating area. A honeycomb metal grid is installed upstream of the flow rectification area to smooth the flow and prevent fish from escaping. After passing through the grid, the water flow develops freely in the rectification area to reduce turbulence and ensure sufficient stability in the test area. A metal grid is also installed downstream of the preparation area to prevent fish from escaping.

Two video cameras (HIKVISION, Hangzhou, China, 18 Hz) are installed 4 m above the flume and connected to a high-definition recorder (HIKVISION, HNVR6108) to record the real-time activity and position of the experimental fish during the trial. To facilitate the identification of the fish in turbulent flow videos, white plastic sheets are laid at the bottom of the flume to enhance the contrast between the fish and the background. A side camera (HIKVISION, 18 Hz) is also installed on the flume to differentiate the primary activity water layer of the fish and record the real-time water depth. The static water depth in the flume is kept constant at 0.30 m. During the experiment, the camera records the real-time depth data marked on the water level gauge, and the flow velocity in the flume is measured every half hour using a propeller (NHRI, LGY-II, Nanjing, China) velocimeter to ensure that both water depth and flow velocity remain constant throughout the experiment.

Three-dimensional hydrodynamic data for the test area is collected using an Acoustic Doppler Velocimeter (ADV) (Nortek As, Rud, Norway). Monitoring sections are set at 0.25 m intervals, with 25 sections in total (numbered from 1 to 25, from downstream to upstream). Each section has 5 measurement points at 0.10 m intervals, totaling 125 measurement points (Figure 1). The ADV’s single-point recording frequency is set to 25 Hz, with a data recording duration of 60 s per point. The monitoring water layer is set at a depth of 0.10 m, as determined by the side-view video monitoring, which shows it to be the layer where the fish are most active. For the central section of the test area, in addition to the 5 measurement points at the 0.10 m depth, flow velocity is also measured at depths of 0.05 m, 0.15 m, and 0.20 m. The average flow velocity at the central monitoring cross section (monitoring cross section 12) is defined as the *V_nom_*, used to characterize the overall flow field’s velocity level (Figure S1).

### 2.2. Fish Husbandry and Trials

The fish used in this study were wild *S. oconnori*, captured using nets on 3 April 2025, in the middle and lower reaches of the Yarlung Tsangpo River near Nyingchi, Tibet. After capture, the fish were transferred to a transport vehicle containing water from the Yarlung Tsangpo River, equipped with a recirculating water flow system and oxygen supply to maintain sufficient oxygen levels. The fish were then transported to the Zangmu fish breeding and release station, where they were kept in two circular breeding tanks (diameter 3.00 m, depth 1.00 m, each equipped with an aeration pump). Before use, the breeding tanks were disinfected with potassium permanganate to minimize disease risks. The breeding water was sourced from the Yarlung Tsangpo River (50% water change daily), and water temperature and dissolved oxygen levels were monitored hourly using an oxygen meter (HACH, LDO10103, Loveland, CO, USA). During the trial period, the water temperature was maintained between 12.21 °C and 13.40 °C, and the dissolved oxygen levels were kept between 6.11 mg/L and 7.04 mg/L. The fish were fed commercial feed at scheduled times each day, up until 24 h before the trial began.

All trials were conducted during the spawning season of *S. oconnori* (March to May) [48], specifically from 12 April to 12 May 2025. Before the trial, the flume was filled with water to a depth of 0.30 m. In each trial, a healthy and active fish was randomly selected and transferred from the breeding tank to the staging area for free swimming. To prevent excessive energy expenditure from long-time swimming during acclimatization (which typically lasts 0.5 to 12 h [49]), the water pumps were turned on after 0.50 h of environmental acclimatization to initiate the test. Stimulated by the flow, the fish began to swim upstream in the test area. When the fish abandoned its attempt to swim upstream and descended back to the staging area, a dip net was used to encourage the fish to continue attempting upstream movement. If after three attempts of netting, the fish showed no further inclination to swim upstream or failed to respond to the netting, it was considered fatigued, and the trial was terminated. The fish was then captured with the net for measurement of body weight and length. Based on the results from enclosed chamber tests, the burst swimming speed of *S. oconnori* ranged from 1.21 m/s to 1.69 m/s [7]. Therefore, for each trial, the pumps were adjusted to a preset power level, and the *V_nom_* was set to one of five levels: 1.80 m/s, 2.00 m/s, 2.20 m/s, 2.40 m/s, and 2.50 m/s. The average body length of the trial fish was 39.16 cm, resulting in corresponding relative flow velocities of 4.60 BL/s, 5.11 BL/s, 5.62 BL/s, 6.13 BL/s, and 6.38 BL/s, respectively. This setup was designed to investigate the swimming performance and behavioral preferences of *S. oconnori* under high-flow conditions (Table 1).

### 2.3. Hydraulic Characteristics

In addition to the directly measured flow velocity (*V_f_*), this study assumes that the factors most influential to fish swimming behavior also include turbulent kinetic energy (*TKE*) and Reynolds shear stress (τ) in each direction. *TKE* is a characteristic value that reflects the intensity of velocity fluctuations in the water flow [50]:

(1) $$TKE=\frac{1}{2}(\frac{1}{n}\sum_{i=1}^{n}{\left(u_{i}-\bar{u}\right)}^{2}+\frac{1}{n}\sum_{i=1}^{n}{\left(v_{i}-\bar{v}\right)}^{2}+\frac{1}{n}\sum_{i=1}^{n}{\left(w_{i}-\bar{w}\right)}^{2})$$

where *n* is the number of samples for each measurement point, $\bar{u}$, $\bar{v}$ and $\bar{w}$ are the time-averaged flow velocities in the longitudinal, lateral, and vertical directions, respectively, and $u_{i}$, $v_{i}$, and $w_{i}$ are the instantaneous flow velocities in the longitudinal, lateral, and vertical directions, respectively.

τ refers to the shear stress generated between two adjacent layers in a plane due to differences in velocity [51]. It is defined as follows, with $\tau_{uv}$, $\tau_{uw}$ and $\tau_{vw}$ calculated in a similar manner:

(2) $$\tau_{uv}=-\rho \frac{1}{n}\sum_{i=1}^{n}\left(u_{i}-\bar{u}\right)(v_{i}-\bar{v})$$

(3) $$\tau_{uw}=-\rho \frac{1}{n}\sum_{i=1}^{n}\left(u_{i}-\bar{u}\right)(w_{i}-\bar{w})$$

(4) $$\tau_{vw}=-\rho \frac{1}{n}\sum_{i=1}^{n}\left(v_{i}-\bar{v}\right)(w_{i}-\bar{w})$$

where *ρ* is the density of water, 10^3^ kg/m^3^.

### 2.4. Swimming Performance

The Logger Pro 3.16 software (Vernier, Beaverton, OR, USA) was used to track and analyze the position of the trial fish in a two-dimensional space. The recorded video was analyzed at a sampling frequency of 18 fps, and the fish’s position coordinates were sequentially connected to extract the individual movement trajectory.

Swimming speed is the most direct indicator of fish swimming performance. First, the swimming speed of the fish relative to the ground ($\vec{V_{g}}=\vec{s}/\Delta t$), where $\vec{s}$ is the displacement between consecutive frames, and Δt is 1/18 s, was calculated. The absolute swimming speed of the fish was then calculated using the vector sum method, based on the measured flow velocity data ($\vec{V_{s}}=\vec{V_{g}}-\vec{V_{f}}$). According to the angle between $\vec{V_{g}}$ and $\vec{V_{f}}$, the fish’s movement mode was classified into downstream movement (0−π/3), cross-stream movement (π/3−2π/3), and upstream movement (2π/3−π) [32].

Endurance, as an important indicator of fish physiological performance, reflects the relationship between covariates and fatigue time (*T*). The prolonged model established in this study aims to quantify the non-sustained swimming performance of fish. Sustained swimming speed refers to the range of swimming speeds that do not lead to fatigue, with endurance fully relying on aerobic metabolic pathways [52]. Approximate values derived from studies on the critical swimming speed of *S. oconnori* suggest that swimming speeds below 3.83 BL/s are generally considered within the sustained swimming range [7]. To minimize the interference of sustained swimming behavior on the prolonged model, this study defines *T* as the total duration during which the trial fish swims at speeds exceeding 3.83 BL/s. The average swimming speed is then calculated as the arithmetic mean of the swimming speeds during this period.

### 2.5. Swimming Behavior

**Figure 2:**
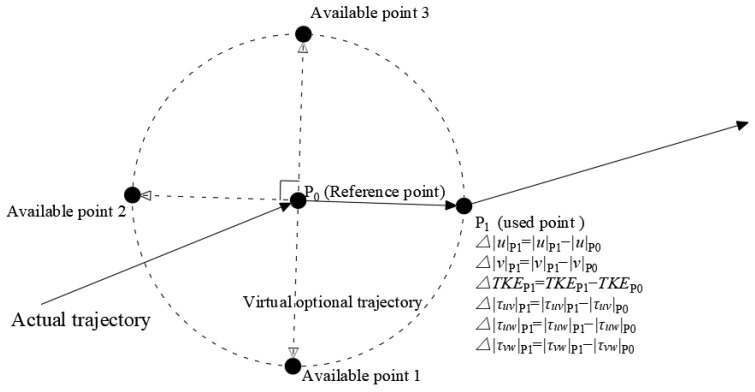
Schematic diagram illustrating the construction of the used-available decision-making dataset for fine-scale movement analysis. The dashed lines and hollow arrows indicate the virtual optional trajectories (from reference point P_0_ to available points 1–3), while the solid lines and solid arrows represent the actual trajectory (from reference point P_0_ to the used point P_1_).

To achieve fine-scale behavioral mechanism analysis, this study constructed a dataset of 34,953 use-availability data points based on single-frame resolution. Specifically, for each dynamic trajectory point (excluding stationary states), the previous frame position P_0_ was used as the reference point, and a circular utilization space was constructed with the Euclidean distance between P_0_ and the subsequent frame position P_1_ as the radius. To address the data balance issue, within this space, three points were selected along the line connecting P_0_ and P_1_ at the angles of π/2 (perpendicular direction), π (reverse direction), and 3π/2 (perpendicular reverse direction), forming a candidate set of available points. This process resulted in a complete decision scenario dataset, including both actual movement trajectories and virtual optional trajectories (Figure 2).

### 2.6. Statistical Methods

#### 2.6.1. Swimming Performance

To statistically analyze the swimming speed of fish, the longitudinal length of test area was divided into intervals of 0.1 m. For each interval, the average values and 95% confidence intervals of *V_s_*, *V_f_* and *V_g_* were calculated during the upstream movement phase A relationship curve was constructed between *V_s_*, *V_f_* and *V_g_*, and movement distance (*x*). Pearson’s correlation coefficient was used to analyze the correlation between different speeds, and the coefficient of variation (CV) was used to analyze the stability of the speed, with a significance level set at 0.01.

The Accelerated Failure Time (AFT) model is used to analyze the correlation between *V_s_*, *BL*, *Temp*, *DO* and *T* [53]. The Akaike Information Criterion (AIC) is employed to select the best-fitting model by minimizing the AIC values across all possible combinations of independent variables. The AFT model, a parametric survival analysis method, is an extension of Cox proportional hazard regression and effectively handles survival analysis problems involving censored data [54]. In this study, individuals that swim a distance exceeding the total length of the test area are recorded as censored data. The structural form of the AFT model is as follows:

(5) $$\ln(T)=\beta_{0}+\beta_{1}C_{ps}+(\beta_{2}+\beta_{3}C_{ps})x_{1}+\cdot \cdot \cdot +(\beta_{2k}+\beta_{2k+1}C_{ps})x_{k}+\varepsilon$$

where *T* is the fatigue time (s); β is the regression coefficient; $x_{k}$ is the *k*-th covariate; and ε is the error term that follows a Weibull distribution. *C_ps_* is a binary variable indicating whether the fish is swimming in the prolonged (0) or sprint (1) mode. The breakpoint is determined using the moving point regression method [55], where the model iteratively runs for each observation, gradually increasing the assumed breakpoint (between prolonged and sprint modes) to match each observed swimming speed. Using this method, all potential breakpoints for the given observational data were tested. The model coefficients are estimated for each case, and the AIC and consistency index (C-index) are calculated to determine which breakpoint best describes the data and provides the optimal discriminative ability of the model.

#### 2.6.2. Swimming Behavior

To further investigate the path selection behavior mechanisms of fish in high-flow environments, this study employs an RSF analysis framework based on Binomial Generalized Additive Mixed Models (GAMMs). As a core statistical tool in ecology for quantifying animal resource use preferences, the RSF compares the resources actually utilized by animals with those available but unused in the environmental background, establishing a quantitative relationship between resource characteristics and selection probability [56]. Given the nonlinearity and randomness in fish behavioral mechanisms, this study introduces GAMMs as the core modeling tool within the above framework. As an extension of the Generalized Additive Model (GAM) with mixed effects, GAMMs combine the advantages of both non-parametric smoothing functions and random effects: on one hand, the smoothing function flexibly captures the complex nonlinear relationships between predictor and response variables, effectively characterizing the nonlinear response patterns in fish behavioral decision-making; on the other hand, the random effects term accounts for individual differences, repeated measurements, and other data structure characteristics, improving the precision of model parameter estimates.

Based on the construction of the use-availability dataset within the RSF framework (Figure 2), and considering the nonlinear characteristics of the relevant mechanisms, this study employs GAMMs to analyze the behavioral selection mechanisms of fish in complex flow environments. All statistical analyses were performed in the R (4.5.1) software environment [57], with model construction based on the mgcv package (1.9-4) [58]. Model diagnostics and visualization were carried out using the gratia package (0.11.1) [59], and the analysis process was conducted within the RStudio (2025.05.1) integrated development environment [60]. The core equation of the GAMMs is as follows:

(6) $$\mathrm{logit}(P)=\beta_{0}+\sum f_{i}(x_{i},by=Mo)+\sum f_{j}(x_{j},E)+s(Mo)+s(fish\text{\_}id)$$

where logit is the logit function, *P* represents the probability of using a specific location (a binary variable, 1 = used point, 0 = unused point); $\beta_{0}$ is the model intercept, *f* represents the spatial autocorrelation (thin plate smoothing function); $x_{i}$ and $x_{j}$ represent $\Delta \left|u\right|$, $\Delta \left|v\right|$, ΔTKE, $\Delta \left|\tau_{uv}\right|$, $\Delta \left|\tau_{uw}\right|$ and $\Delta \left|\tau_{vw}\right|$, defined as spatial intensity differences in hydraulic factors—i.e., the absolute difference between the hydraulic factor at an available point and at the fish’s reference point—used to quantify instantaneous decision-making based on local environmental contrasts (Figure 2). *Mo* represents the movement mode chosen by the fish (upstream, downstream, or cross-stream movements); *E* is the endurance state, which refers to the remaining percentage of swimming endurance in fish under voluntary conditions, defined as $E=1-\int_{t=0}^{t_{*}}T^{-1}dt$, where *T* is obtained from Equation (5) and $t_{*}$ is the movement duration [61]. $\sum f_{i}(x_{i},by=Mo)$ are conditional smooth terms for $x_{i}$ stratified by *Mo*, used to capture behavior rules under different *Mo*. $\sum f_{j}(x_{j},E)$ are interaction smooths between $x_{j}$ and *E*, representing the moderating effect of endurance on selection behavior. s(Mo) and s(fish_id) are random-effect terms controlling inherent differences among movement modes and among individual fish, respectively.

The basis function dimension for each smoothing term is set between 3 and 5, with the final smoothness controlled by the smoothing parameter automatically estimated using the fast restrictive maximum likelihood method to prevent overfitting. Prior to modeling, Pearson correlation analysis (r > 0.6) and variance inflation factor (VIF > 3) tests were conducted to check for multicollinearity among variables. Model selection was performed using the AIC, and the model was evaluated using 10-fold cross-validation. To further explore the nonlinear effects and interactions of key driving factors on the behavioral decisions of *S. oconnori* in high-flow environments, this study uses partial dependence plots (PDP) to analyze univariate key response thresholds and bivariate interactions [62].

## 3. Results

### 3.1. Hydrodynamic Characteristics of the Flume

**Figure 3:**
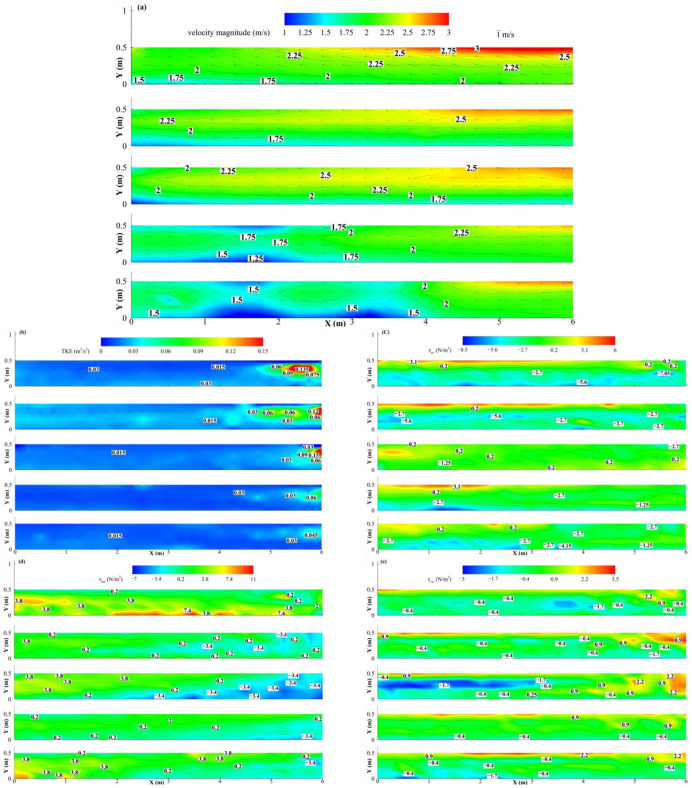
Contour maps of hydraulic factors in the test area, with *V_nom_* decreasing progressively from top to bottom in a single image: (**a**) *V_f_* (m/s), with black arrows indicating the direction of flow velocity, and their length representing flow velocity magnitude. White labels show the values represented by each contour line. (**b**) *TKE* (m^2^/s^2^), (**c**) $\tau_{uv}$ (N/m^2^), (**d**) $\tau_{uw}$ (N/m^2^), (**e**) $\tau_{vw}$ (N/m^2^).

*V_nom_* is a key driving factor controlling the flow velocity and its spatial distribution within the flume. As shown in Figure 3a, the flow field distributions under five different *V_nom_* conditions systematically reveal their significant impact on the flow field structure, from high to low. When *V_nom_* is 2.50 m/s, the flow field generally exhibits high-energy characteristics, with higher flow velocities in the main flow region. The maximum flow velocity of 3.20 m/s is observed in the right bank area of the test area. As *V_nom_* decreases to 2.40 m/s, the energy state of the flow field weakens accordingly, with the maximum flow velocity decreasing to 2.84 m/s. The high-flow region shrinks significantly, while the proportion of medium and low-flow regions increases. The flow velocity vector arrows also gradually become smoother, indicating a reduction in the turbulence of the overall flow structure. In the intermediate conditions (*V_nom_* = 2.20 m/s and 2.00 m/s), the flow field further transitions to a medium-speed range, with a more uniform flow pattern. When *V_nom_* reaches the lowest value of 1.80 m/s, a fundamental change occurs in the flow field, with low-flow velocities dominating. The minimum flow velocity is approximately 0.80 m/s, and the flow pattern becomes smoother and more stable. These results consistently indicate that a decrease in *V_nom_* directly leads to a reduction in flow field energy, a shrinking of the high-velocity area, and a shift toward a more uniform and stable flow pattern.

The intensity and distribution pattern of *TKE* show significant dependence on the flow conditions. Figure 3b illustrates the spatial distribution of *TKE* under different *V_nom_* conditions. Under all conditions, the maximum *TKE* values are concentrated at the upstream end of the right bank of the test area. Comparing with Figure 3a, it is evident that the high *TKE* region is concentrated in areas with high flow velocity spatial intensity differences (i.e., where the contour lines are most densely packed), indicating that this region is where the dissipation of endurance and turbulent mixing is most intense in the flow. Additionally, *TKE* is highly sensitive to changes in overall flow intensity. As *V_nom_* decreases from 2.50 m/s to 1.80 m/s, the *TKE* intensity systematically decreases. When *V_nom_* = 2.50 m/s, the maximum *TKE* reaches 0.30 m^2^/s^2^. As the flow velocity decreases, the high *TKE* region significantly shrinks, and its intensity weakens. The main flow area is gradually replaced by medium and low *TKE* regions. When *V_nom_* drops to 1.80 m/s, the maximum *TKE* value is only 0.05 m^2^/s^2^, indicating that the flow has become smoother and the turbulence intensity has significantly decreased.

Figure 3c–e show the spatial distribution of the three Reynolds shear stress components, $\tau_{uv}$, $\tau_{uw}$, and $\tau_{vw}$, under different nominal flow velocities. The high-intensity regions of Reynolds shear stress exhibit systematic spatial distribution patterns. The larger values of the three Reynolds shear stress components are consistently and significantly distributed near the two side walls of the channel, reflecting strong shear interactions between fluid particles and the wall surfaces. The specific distribution patterns of different stress components show differences, revealing the anisotropic characteristics of the flow structure. The high-value region of $\tau_{uv}$ forms a continuous band along the wall, clearly outlining the boundary of momentum transport in the mainstream flow. As *V_nom_* gradually decreases, the maximum absolute value of $\tau_{uv}$ decreases from 7.55 N/s^2^ to 7.40 N/s^2^. Meanwhile, the distribution of $\tau_{uw}$ and $\tau_{vw}$ displays stronger local patch-like structures, suggesting the complexity of vertical momentum distribution. Vertical momentum transport is significantly influenced by local vortex structures. The absolute value distribution of the two variables is not strongly correlated with *V_nom_*. As *V_nom_* decreases, the maximum absolute value of $\tau_{uw}$ drops from 11.19 N/s^2^ (at *V_nom_* = 2.50 m/s) to 4.03 N/s^2^ (at *V_nom_* = 2.40 m/s), and then gradually increases to 9.20 N/s^2^ (at *V_nom_* = 1.80 m/s). The maximum absolute value of $\tau_{vw}$ increases from 2.88 N/s^2^ (at *V_nom_* = 2.50 m/s) to 3.35 N/s^2^ (at *V_nom_* = 2.40 m/s), and then gradually decreases to 2.87 N/s^2^ (at *V_nom_* = 1.80 m/s).

### 3.2. Swimming Performance

#### 3.2.1. Swimming Speed

**Figure 4:**
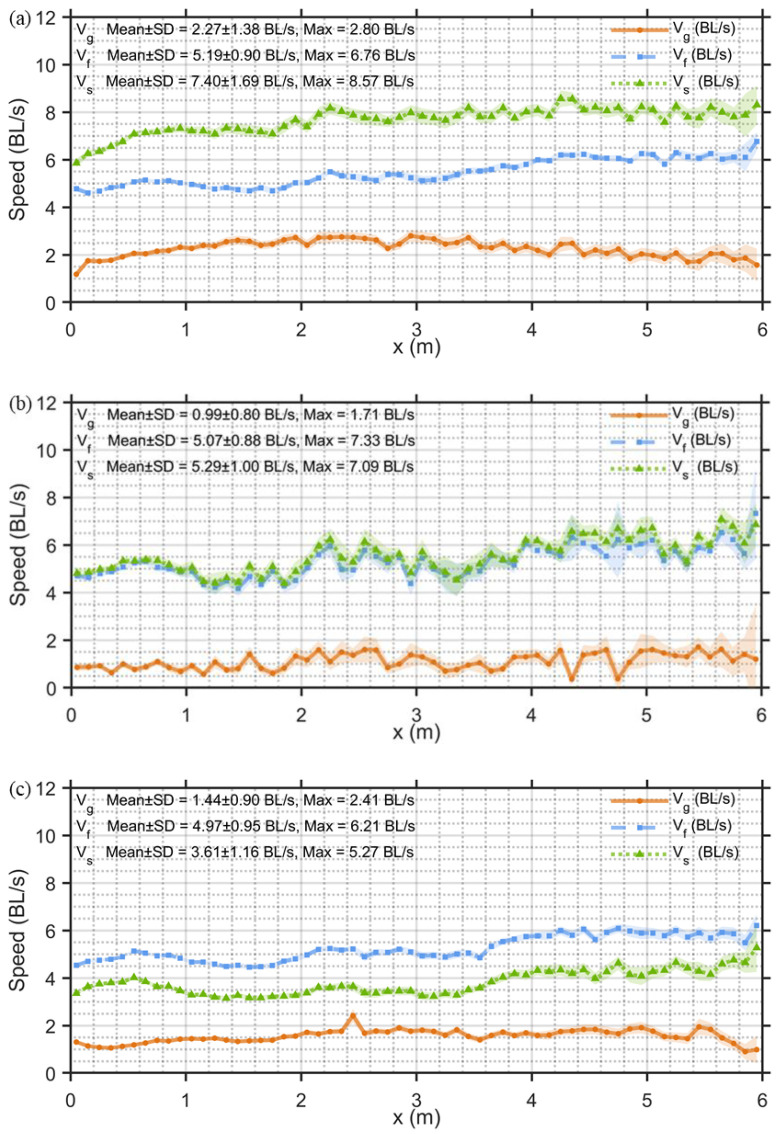
The relationship between *V_g_*, *V_f_*, *V_s_*, and movement distance (*x*) with the average and 95% confidence intervals, using length-adjusted units (based on body length). The data are presented for five *V_nom_*, considering both the fish’s trajectory and the spatial variation in water speed, for different *Mo*: (**a**) Upstream movement, (**b**) Cross-stream movement, (**c**) Downstream movement.

Based on the results shown in Figure 4, the relationship between *V_g_*, *V_f_* and *V_s_* with respect to movement distance (*x*) was analyzed for the three *Mo*: upstream movement, cross-stream movement, and downstream movement. Overall, *V_f_* increases gradually with *x* in all modes, reflecting the spatial distribution characteristics of *V_f_* within the test area (Figure 3a).

The speed parameters of fish exhibit significant differences under different *Mo*. In the upstream movement mode (Figure 4a), *V_s_* increases from the initial position and stabilizes at 7.96 BL/s after 2.0 m (CV = 3.22%), with an overall average value of 7.40 BL/s. *V_g_* shows an increasing and then decreasing trend, as *V_s_* stabilizes and *V_f_* continues to increase (with an average value of 5.19 BL/s), with a mean value of 2.27 BL/s. In the cross-stream movement mode (Figure 4b), the development trends of *V_s_* and *V_f_* are highly consistent (r = 0.97, *p* < 0.001) and similar in magnitude (*V_s_* average value is 5.29 BL/s, *V_f_* average value is 5.07 BL/s), resulting in a lower *V_g_* that maintains a relatively steady level, with an average value of 0.99 BL/s. In the downstream movement mode (Figure 4c), the fish apply a swimming speed (*V_s_* average value of 3.61 BL/s) opposite to the flow direction to counteract some of the flow’s push (*V_f_*, average value of 5.05 BL/s). *V_s_* and *V_f_* maintain a relatively consistent trend (r = 0.93, *p* < 0.001), thus achieving relatively stable *V_g_*, with an average value of 1.44 BL/s.

#### 3.2.2. Endurance

For the swimming endurance study of *S. oconnori*, a total of 15 candidate models were constructed based on various combinations of candidate covariates. Table 2 presents the three models with the lowest AIC values. The results indicate that *V_s_* and *BL* are the primary predictors of swimming endurance in *S. oconnori*, while *Temp* and *DO do* not significantly affect endurance (*p* > 0.05).

**Figure 5:**
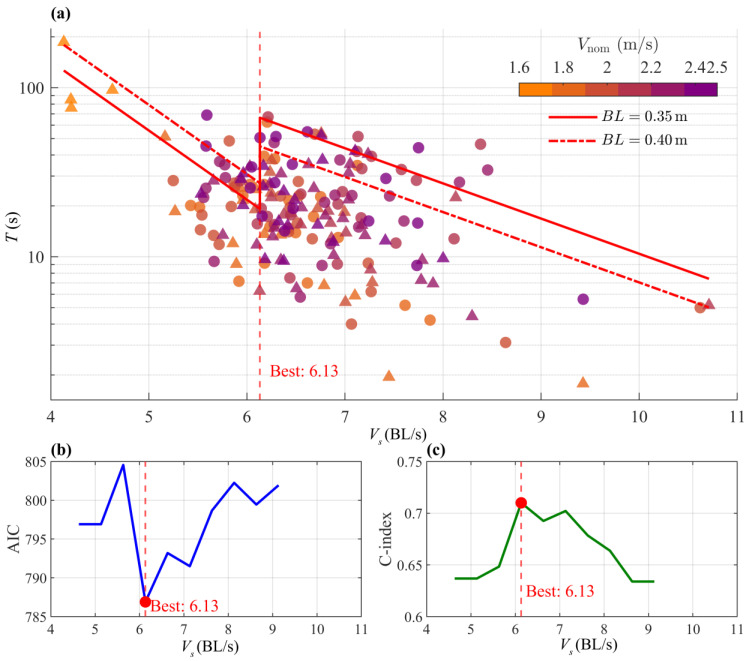
(**a**) The relationship between *V_s_*, *BL* and *T* for *S. oconnori*. Swimming speeds less than 3.83 BL/s were excluded from the model. *BL* (in millimeters) is represented by different line styles: solid line for a fish of 0.35 m length and dashed line for a fish of 0.40 m length. *V_nom_* is represented by a color gradient. The prolonged-sprint transition occurs around 6.13 BL/s. Censored results are shown as circles, while fully (fatigue) observed results are shown as triangles. The changes in AIC and C-index values are shown in panels (**b**,**c**), the red dots indicates the values of *V_s_* that minimize the AIC or maximize the C-index, respectively, serving as the selection criteria for the optimal model.

*T* decreases with increasing *V_s_*, and there is clear evidence of a transition between the prolonged mode and sprint mode around 6.13 BL/s (Table 3 and Figure 5). At this point, the model’s AIC reaches its minimum value, and the consistency index (C-index) reaches its maximum value of 0.71, indicating good discriminative ability. The body size effect is specific: at the same *V_s_*, larger *S. oconnori* exhibit greater endurance in the prolonged mode compared to smaller individuals, while smaller fish perform better in the sprint mode. This means that, compared to a 0.35 m long *S. oconnori*, a 0.40 m long fish has 1.4 times the endurance in the prolonged mode and 0.68 times the endurance in the sprint mode when swimming at the same speed (Table 3 and Figure 5).

### 3.3. Swimming Behavior

#### 3.3.1. Relationship Between Behavioral Strategies and Movement Mode

After conducting correlation and collinearity tests, all indicators met the required criteria (Figure S2). The indicators included in the RSF-GAMMs for *S. oconnori*’s hydraulic preference are shown in Table S1. All smooth terms for spatial intensity differences in environmental variables grouped by *Mo*, as well as all interaction smooth terms between spatial intensity differences in environmental hydraulic factors and *E* (except for $\Delta \left|\tau_{uv}\right|$), were identified as key driving factors for fish path selection. The average AUC value from the 10-fold cross-validation was 0.732, with a recall rate of 0.719 and a balanced accuracy of 0.672, indicating that the model can effectively explain the relationship between *S. oconnori*’s path selection behavior under high-flow conditions and various hydraulic factors, movement modes, and endurance states.

**Figure 6:**
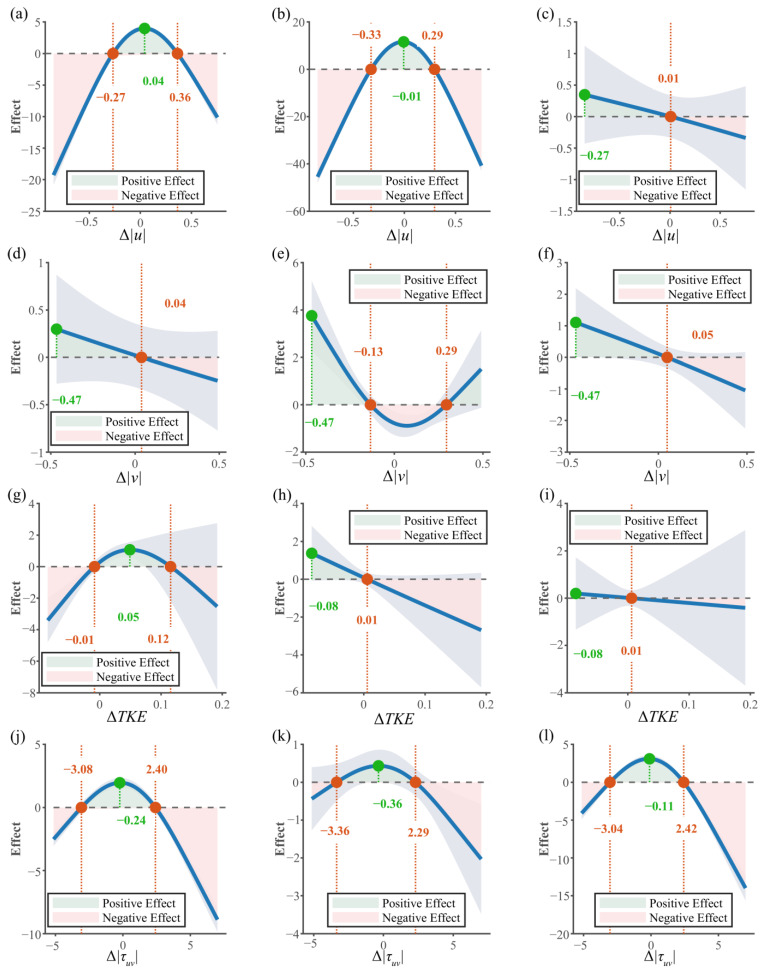

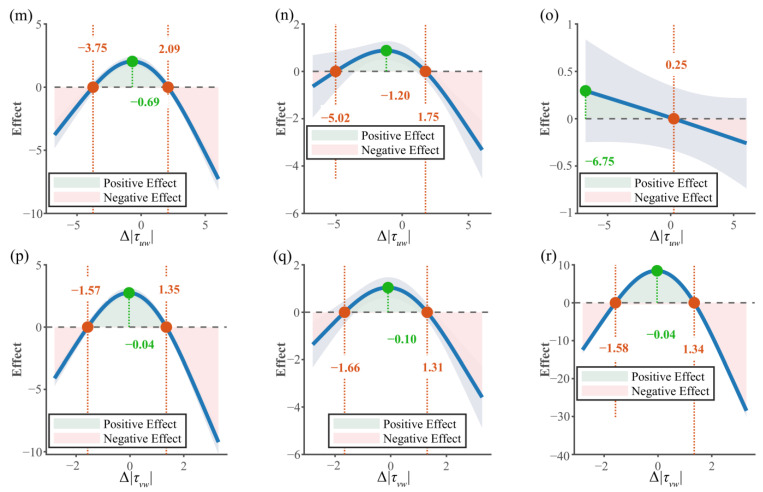
Key points showing the relationship between different hydraulic factors’ spatial intensity differences under various *Mo*. The shaded area represents the 95% confidence intervals. The green dot indicates the covariate value corresponding to the maximum positive effect, while the red dot marks the covariate value where the effect equals zero. Panels (**a**–**c**), (**d**–**f**), (**g**–**i**), (**j**–**l**), (**m**–**o**), and (**p**–**r**) represent $\Delta \left|u\right|$, $\Delta \left|v\right|$, ΔTKE, $\Delta \left|\tau_{uv}\right|$, $\Delta \left|\tau_{uw}\right|$, $\Delta \left|\tau_{vw}\right|$, respectively. (**a**) Upstream movement $\Delta \left|u\right|$; (**b**) Cross-stream movement $\Delta \left|u\right|$; (**c**) Downstream movement $\Delta \left|u\right|$; (**d**) Upstream movement $\Delta \left|v\right|$; (**e**) Cross-stream movement $\Delta \left|v\right|$; (**f**) Downstream movement $\Delta \left|v\right|$; (**g**) Upstream movement ΔTKE; (**h**) Cross-stream movement ΔTKE; (**i**) Downstream movement ΔTKE; (**j**) Upstream movement $\Delta \left|\tau_{uv}\right|$; (**k**) Cross-stream movement $\Delta \left|\tau_{uv}\right|$; (**l**) Downstream movement $\Delta \left|\tau_{uv}\right|$; (**m**) Upstream movement $\Delta \left|\tau_{uw}\right|$; (**n**) Cross-stream movement $\Delta \left|\tau_{uw}\right|$; (**o**) Downstream movement $\Delta \left|\tau_{uw}\right|$; (**p**) Upstream movement $\Delta \left|\tau_{vw}\right|$; (**q**) Cross-stream movement $\Delta \left|\tau_{vw}\right|$; (**r**) Downstream movement $\Delta \left|\tau_{vw}\right|$.

PDP were used to quantify the specific effects of different hydraulic factors’ spatial intensity differences on path selection by *S. oconnori* under various *Mo*. Positive effect values represent an enhancing effect on the probability of selecting a particular spatial location, while negative values indicate a suppressive effect (Figure 6).

The response of *S. oconnori* to $\Delta \left|u\right|$ shows significant differences across the three movement modes: upstream, cross-stream, and downstream (Figure 6a–c). In the upstream and cross-stream modes, the fish exhibit a preference for values close to zero, with positive effect ranges of −0.27 m/s to 0.36 m/s and −0.33 m/s to 0.29 m/s, and optimal effect points at 0.04 m/s and −0.01 m/s, respectively. However, in the downstream mode, the response curve shifts leftward, with the positive effect range concentrated in the negative value region (−0.85 m/s to 0.01 m/s), and the optimal effect point located at −0.85 m/s.

For the response to $\Delta \left|v\right|$ (Figure 6d–f), a negative monotonic decreasing trend is observed in both the upstream and downstream modes (Figure 6d–f). The optimal effect values in these two modes are both located at −0.47 m/s, with the positive effect ranges being −0.47 m/s to 0.04 m/s (upstream) and −0.47 m/s to 0.05 m/s (downstream). In the cross-stream mode (Figure 6e), the response curve takes a U-shape, with the positive effect range concentrated in the regions $\Delta \left|v\right|$ < −0.13 m/s and $\Delta \left|v\right|$ > 0.29 m/s. When $\Delta \left|v\right|$ is between −0.13 m/s and 0.29 m/s, the effect is negative.

The response to ΔTKE (Figure 6g–i) varies depending on the movement mode. In the upstream mode, the relationship follows an inverted U-shape, with the positive effect range being −0.01 m^2^/s^2^ to 0.12 m^2^/s^2^, and the optimal effect point at 0.05 m^2^/s^2^. In contrast, both the cross-stream and downstream modes show a negative monotonic decreasing trend, with the positive effect range concentrated in the region ΔTKE < 0.01 m^2^/s^2^, and the optimal effect point at −0.08 m^2^/s^2^ in both modes.

The response to the differences in Reynolds shear stress components ($\Delta \left|\tau_{uv}\right|$, $\Delta \left|\tau_{uw}\right|$, $\Delta \left|\tau_{vw}\right|$) is shown in Figure 6j–r. For $\Delta \left|\tau_{uv}\right|$ (Figure 6j–l), all three movement modes exhibit an inverted U-shape relationship. The positive effect ranges are similar across the modes: upstream movement is −3.08 N/m^2^ to 2.40 N/m^2^, cross-stream movement is −3.36 N/m^2^ to 2.29 N/m^2^, and downstream movement is −3.04 N/m^2^ to 2.42 N/m^2^. The optimal effect values are slightly below zero, at −0.24 N/m^2^, −0.36 N/m^2^, and −0.11 N/m^2^, respectively. For $\Delta \left|\tau_{uw}\right|$ (Figure 6m–o), the upstream and cross-stream modes show an inverted U-shape relationship, with the optimal effect points at −0.69 N/m^2^ and −1.20 N/m^2^, respectively. The downstream mode shows a trend closer to a negative monotonic decrease, with the optimal effect value at −6.75 N/m^2^. For $\Delta \left|\tau_{vw}\right|$ (Figure 6p–r), all modes exhibit an inverted U-shape relationship, with similar positive effect ranges (−1.6 N/m^2^ to 1.3 N/m^2^). The optimal effect values are close to zero, at −0.10 N/m^2^ (upstream), and −0.04 N/m^2^ (cross-stream and downstream).

#### 3.3.2. Interaction Between Endurance State and Hydraulic Factors

**Figure 7:**
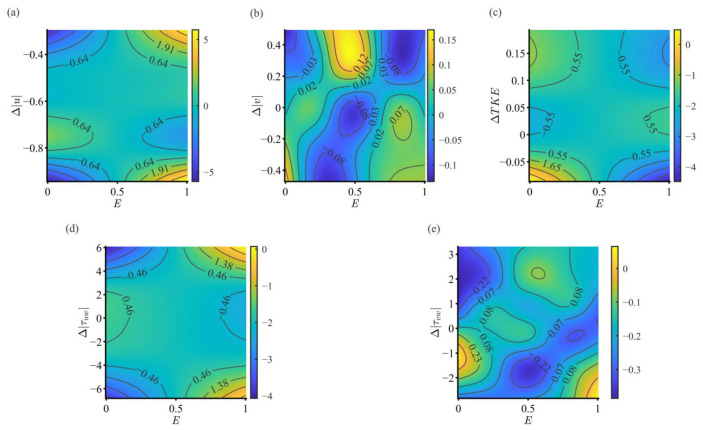
Interaction between *E* and spatial intensity differences in various hydraulic variables for *S. oconnori*. (**a**) Partial dependence of *E* and $\Delta \left|u\right|$, (**b**) Partial dependence of *E* and $\Delta \left|v\right|$, (**c**) Partial dependence of *E* and ΔTKE, (**d**) Partial dependence of *E* and $\Delta \left|\tau_{uw}\right|$, (**e**) Partial dependence of *E* and $\Delta \left|\tau_{vw}\right|$.

In this study, all interaction smooth terms between environmental hydraulic variables’ spatial intensity differences (except for $\Delta \left|\tau_{uv}\right|$) and *E* were identified as key driving factors for fish path selection. PDP were used to analyze the interaction between *E* and the spatial intensity differences in each hydraulic variable (Figure 7a–e).

Figure 7a illustrates the effect of the interaction between $\Delta \left|u\right|$ and *E* on selection behavior. At high-endurance states (*E* is close to 1), the fish exhibit strong selectivity, and the positive effect is mainly concentrated in the areas where the absolute value of $\Delta \left|u\right|$ is relatively large (i.e., the upper right and lower right corners of Figure 7a). However, at low-endurance states (*E* is close to 0), the fish’s selection strategy changes, showing a strong negative effect in the areas where the absolute value of *E* is significantly large.

Figure 7b illustrates the effect of the interaction between $\Delta \left|v\right|$ and *E*. When fish are in high and low endurance states, the positive effect appears in the negative region of $\Delta \left|v\right|$; at a medium endurance state, the most significant positive effect appears in the positive region of $\Delta \left|v\right|$.

Figure 7c illustrates the effect of the interaction between ΔTKE and *E*. The positive selection preference of fish in a high endurance state is mainly concentrated in the negative region where the absolute value of ΔTKE is relatively small. At low-endurance states, the strongest positive selection preference of the herring is concentrated in the negative region of ΔTKE. This indicates a clear risk-averse, energy-saving strategy. Fish in a low-endurance state will actively seek and select water bodies where the *TKE* is significantly lower than their current location, maximally reducing water turbulence and unstable swimming corrections, thereby reducing energy consumption.

The interactive effects of the Reynolds shear stress differences $\Delta \left|\tau_{uw}\right|$ and $\Delta \left|\tau_{vw}\right|$ with *E* in Figure 7d,e collectively reveal the regulatory effect of *E* on the fish’s strategy for selecting water bodies with vertical momentum exchange differences. The positive selection effects of the herring in a high endurance state on $\Delta \left|\tau_{uw}\right|$ and $\Delta \left|\tau_{vw}\right|$ appear in both extreme negative and extreme positive regions, indicating that fish can switch strategies during periods of sufficient endurance. Fish in a low-endurance state chooses to respond in regions where the absolute values of $\Delta \left|\tau_{uw}\right|$ and $\Delta \left|\tau_{vw}\right|$ are relatively small, suggesting that fish with subpar endurance state tend to shift towards a conservative approach, inclining to select water bodies with relatively stable or smaller Reynolds shear stresses to ensure survival.

## 4. Discussion

### 4.1. Movement Mode Dependency of Swimming Speed

This study quantifies the swimming speed of *S. oconnori* under three typical movement modes (upstream movement, cross-stream movement, downstream movement) in an open-channel flume under high-flow conditions, revealing a core finding: the magnitude and variation pattern of the swimming speed of *S. oconnori* are significantly regulated by the movement mode and the local water flow environment.

In the upstream movement mode, after an initial acceleration phase, the swimming speed of the fish stabilizes after approximately 2.0 m of movement, forming a distinct “speed plateau” phenomenon, maintaining a relatively high and steady level. This strategy contrasts with the “constant optimal ground swimming speed” theory, which suggests that fish prioritize maintaining a constant ground speed to maximize upstream distance [55]. One possible explanation for this phenomenon is that *S. oconnori* adopts a time-saving strategy when facing high-flow environments [35], swimming through the high-flow water at its maximum speed. Therefore, the plateau swimming speed (*V_s_* = 7.96 BL/s) may represent the fish’s maximum swimming speed, which is significantly higher than the burst swimming speed (3.93 BL/s) measured for similar body length *S. oconnori* in an enclosed swim chamber [7]. This highlights the advantages of the near-natural open-channel flume, which provides sufficient swimming space and diverse flow conditions for the fish [63,64], creating varied hydrodynamic microenvironments due to wall effects. Fish can save energy through mechanisms such as “flow refuging” and “vortex capture” in these environments [65]. In contrast, the limited space and uniform flow conditions in enclosed swim chambers restricts the fish’s performance, preventing them from effectively employing energy-saving strategies, even leading to behavioral fatigue where the fish may actively choose to stop swimming [66].

In the cross-stream movement mode, *S. oconnori* exhibits a typical “flow velocity matching” strategy. Its swimming speed and encountered flow velocity not only show highly consistent trends, but their average values are also very close. This behavioral strategy results in the fish’s ground speed being maintained at a relatively low level (with an average of 0.99 BL/s). In complex flow fields, fish engage in cross-stream movement to explore and select better movement paths [36]. The “flow velocity matching” observed here can be understood as an energy optimization mechanism: by adjusting its swimming speed to match the local flow velocity, the fish minimizes the energy cost of resisting the flow and achieving lateral displacement [41]. This allows the fish to allocate more energy resources to tasks such as environmental perception, path decision-making, and position control, reflecting its adaptive mechanism for efficient movement in dynamic hydrodynamic environments [67].

Finally, in the downstream movement mode, *S. oconnori*’s behavior is not passively “drifting with the current”. Its swimming speed is directed opposite to the flow, and its variation closely matches that of the encountered flow velocity This active “braking” behavior, where the fish applies a reverse speed, effectively counteracts some of the water flow’s pushing force, keeping its ground speed relatively stable at a lower level. The downstream movement behavior observed in this study is characterized by active “braking” and the fish swimming head-up, which suggests that it is more likely a passive compromise or position adjustment strategy after failed upstream movement [68,69], rather than an active downstream migration strategy. This behavior helps fish actively control their position and movement trajectory in turbulent water flows in natural rivers, which is significant for behaviors such as avoiding obstacles, seeking refuge from flow velocity, or preparing for upstream movement [70]. Therefore, from both energy expenditure and behavioral intent perspectives, this is markedly different from the actively described downstream behavior in the literature [37,38,39].

From an energy allocation perspective, this study reveals the dual impact of flow velocity barriers on the energy consumption of upstream migrating fish: not only does continuous opposition to the flow during upstream movement lead to significant energy expenditure, but even during downstream movement, the “braking” behavior implemented by the fish to control its movement state also causes substantial secondary energy consumption. This may exacerbate the fish’s energy depletion and fatigue levels, potentially leading to death [71,72,73]. This finding deepens the understanding of energy trade-off strategies in fish in continuous flow velocity gradient environments and provides important insights for optimizing fish passage facility designs, alleviating energy stress in fish, and conducting habitat restoration research.

### 4.2. High-Altitude Adaptations of S. oconnori’s Endurance

The endurance breakpoint observed at approximately 6.13 BL/s aligns with classical theories in fish swimming physiology [74,75]. When the swimming speed is below the endurance breakpoint, *S. oconnori* is in the prolonged mode, primarily relying on a mixed aerobic-anaerobic metabolism, allowing for prolonged activity. However, when swimming speed exceeds the endurance breakpoint, the fish enters sprint mode, rapidly activating the anaerobic metabolism system, leading to the rapid accumulation of fatigue substances, which causes a dramatic reduction in endurance time. The specific impact of body length on endurance reflects the allometric growth relationship between body size and swimming performance in fish [53,63]. In the prolonged mode, larger individuals possess greater absolute muscle mass and higher energy reserves, providing a solid physiological foundation for sustaining prolonged aerobic activity. In contrast, in sprint mode, smaller individuals have a smaller drag area, reducing resistance during high-speed swimming, allowing them to maintain sprints more effectively [76].

Due to the limited data available from testing *S. oconnori* using the same method, the results of this study are compared with those of other species from different taxonomic groups tested using similar methods [13,55,77]. The intercept of the prolonged mode for *S. oconnori* (6.29) is slightly lower than the historical mean (6.9 ± 1.7), and the absolute value of the slope (−0.94) is much higher than the historical mean (−0.40 ± 0.26). The intercept of the sprint mode (4.48) is very close to the historical mean (4.33 ± 1.14), with no significant difference, while the absolute value of the slope (−0.48) is three times the historical mean (−0.16 ± 0.08). This indicates that not only in the prolonged mode but also in the sprint mode, *S. oconnori* shows a much higher sensitivity to swimming speed than other fish species. *S. oconnori* exhibits very high absolute slope values in both swimming modes, collectively outlining the overall characteristics of its swimming strategy: endurance is highly sensitive to changes in swimming speed, meaning it is relatively not tolerant to high speeds. Whether in prolonged swimming or sprint swimming, increasing swimming speed rapidly depletes endurance time. This characteristic may be an ecological adaptation to the cold, low-oxygen, and highly variable flow conditions of high-altitude rivers [45]. In such environments, fish that are in a hypoxic state may be more inclined to develop strong burst capabilities to cope with instantaneous rapid flows, rather than maintaining high-speed swimming over long durations. The steep slope may indicate that the transition from aerobic to anaerobic metabolism is more rapid and complete, with anaerobic metabolism providing high energy efficiency but very short duration [78].

### 4.3. Navigation Mechanism Based on Energy Conservation and Movement Stability Objectives

This study shows that fish movement decisions are a dynamic process. Under high-flow conditions, the primary goals of *S. oconnori* include minimizing energy consumption and maximizing movement stability. The fish selectively choose water with different levels of disturbance based on their movement mode, optimizing their path selection accordingly.

The response pattern of *S. oconnori* to $\Delta \left|u\right|$ clearly demonstrates the context-dependent nature of their energy-saving strategy. During the upstream and cross-stream modes, where active resistance to flow is required, fish show a preference for flow velocities close to zero, indicating that under high-flow conditions, fish no longer simply seek significant flow gradients for directional navigation but rather prefer relatively stable environmental flow speeds, which helps conserve their energy. The strong preference for significantly negative $\Delta \left|u\right|$ values observed in the downstream mode suggests that fish actively recognize and utilize flow velocity shear and flow sheltering effects during downstream movement to reduce energy expenditure. During longitudinal movement (upstream/downstream), the fish’s monotonic preference for negative $\Delta \left|v\right|$ values can be interpreted as a strategy to suppress unnecessary lateral oscillations, ensuring stability in their movement trajectory. The unique U-shaped preference observed during the cross-stream mode suggests that *S. oconnori* dynamically adjust its strategy based on the relationship between its body orientation and flow direction v to achieve energy-efficient lateral displacement (preferring higher v when aligned with flow and lower v when against it).

The fish’s preference for flow turbulence characteristics also reflects their energy-saving and movement stability mechanisms. When swimming upstream, fish prefer small positive ΔTKE values, whereas during the cross-stream and downstream modes, they consistently avoid high *TKE* regions. This suggests that during the upstream mode, fish may assist their movement by mechanisms such as “vortex capture” from moderate turbulence [79]. In non-upstream modes, fish exhibit a clear avoidance of the energy-consuming effects of water flow turbulence [80].

The balance between the hydrodynamic forces acting on the fish and the resulting torque and moments presents a challenge to maintaining posture and smooth swimming [81]. In all movement modes, fish show a consistent preference for low Reynolds shear stress environments, indicating that they aim to minimize the impact of shear in the flow field on their bodies. This suggests that, regardless of the movement task, maintaining the stability of body posture and controllability of movement direction is a fundamental aspect of their movement strategy [82].

### 4.4. Regulatory Role of Endurance State in Movement Strategy

This study uses PDP to deeply analyze the interaction between the endurance state of *S. oconnori* and its preference for complex hydraulic environments. The results show that the fish’s endurance state is a key regulator of its movement strategy, allowing it to dynamically balance the competing goals of “energy acquisition/efficiency maximization” and “risk avoidance/survival priority” based on its physiological condition.

As endurance state decreases, the behavior of *S. oconnori* shifts continuously from “active exploration” to “conservative risk avoidance.” High endurance states increase the tendency of individuals to select areas with significant hydraulic features. This suggests that when physically strong, the fish have both the capability and willingness to utilize or challenge complex flow structures, possibly aiming for rapid displacement or exploring efficient propulsion modes [83]. This “exploratory” or “opportunistic” strategy aligns with the predictions of optimal foraging theory, where individuals with abundant energy are willing to take on higher risks to gain greater rewards. Conversely, individuals in low endurance states exhibit a strong avoidance tendency, generally steering clear of areas with high hydraulic fluctuations and instead preferring environments with gentler hydraulic gradients [84,85].

Further, from a physiological mechanism perspective, changes in endurance state are directly associated with the redistribution of energy metabolism and immune resources. Individuals in high endurance states can maintain a higher metabolic rate to support intense activity, while in low endurance states, depletion of energy reserves shifts the metabolic burden toward survival functions, resulting in a behavior that reduces non-essential activities [86]. This metabolic trade-off is often accompanied by adjustments in immune strategies—prolonged, high-intensity swimming activates stress responses, and resources may be prioritized for maintaining basic immune functions [87]. As seen in *Procypris rabaudi*, where immune parameters improve after exercise training [88], and in *Siniperca chuatsi*, where the activation of the MAPK pathway alleviates oxidative damage under flow stress [89], these examples demonstrate that fish optimize the distribution of energy between swimming performance, immune defense, and oxidative balance based on their physical condition [90].

## 5. Conclusions

This study aims to describe and quantify the autonomous swimming performance and behavioral strategies of *S. oconnori* in high-flow environments. The research found that its swimming performance significantly exceeded the expectations of traditional enclosed-chamber tests and exhibited strong movement mode dependence: in upstream movement, it adopts a “constant swimming speed” time-saving strategy; in cross-stream movement, it employs a “flow velocity matching” energy optimization strategy; while in downstream movement, it achieves posture control through active “braking”. Endurance analysis revealed a mode breakpoint at approximately 6.13 BL/s, with a specific body size effect. The high sensitivity of endurance to changes in movement intensity highlights its ecological adaptation to the turbulent conditions of high-altitude rivers. In high-flow environments, adult *S. oconnori*’s swimming behavior primarily reflects two main objectives: energy conservation and stability control. The fish selectively choose water with different levels of disturbance based on their movement mode and endurance state, optimizing their path selection accordingly.

The study suggests that constructing hydraulically heterogeneous microhabitats could meet the ecological needs of individuals with different body lengths, movement modes, and endurance states. Future research should combine field validation to further explore the regulatory role of individual experience and group behavior in migration strategies, aiming to promote the development of predictive ecological models and optimize river connectivity restoration.

## Figures and Tables

**Table 1:** Flow velocity treatments, environmental parameters, and fish physiological characteristics in the experiment. *N* refers to the number of fish in each trial, *BL* refers to the body length, *Weight* refers to the body weight, *Temp* refers to the water temperature during the trial, *DO* refers to the dissolved oxygen concentration in the water during the trial, and *Q* refers to the flow rate in the flume.

Trial	*N*	*BL* (Mean ± SD, cm)	*Weight* (Mean ± SD, g)	*Temp* (Mean ± SD, °C)	*DO* (Mean ± SD, mg/L)	*V_nom_* (m/s)	*Q* (m^3^/s)
1	42	38.80 ± 4.57	939.58 ± 327.20	12.70 ± 0.80	6.19 ± 0.07	1.80	0.24
2	41	39.55 ± 4.72	1021.42 ± 373.49	12.89 ± 0.85	6.18 ± 0.06	2.00	0.27
3	40	38.81 ± 4.93	989.08 ± 368.02	12.75 ± 0.80	6.18 ± 0.06	2.20	0.28
4	40	39.66 ± 4.01	992.70 ± 304.00	12.93 ± 0.80	6.20 ± 0.06	2.40	0.28
5	40	38.95 ± 3.96	940.45 ± 320.70	12.94 ± 0.80	6.17 ± 0.05	2.50	0.29

**Table 2:** Prolonged model selection based on AIC.

Rank	Covariates	AIC	ΔAIC	*w*
1	*V_s_* + *BL*	802.62	0	0.64
2	*V_s_* + *BL* + *DO*	804.50	1.88	0.25
3	*V_s_* + *BL* + *DO* + *Temp*	806.24	3.61	0.11

**Table 3:** Models of *V_s_* (using length-adjusted units), *BL*-*T* relationships for prolonged- and sprint-swimming speeds. *V_s_* ranging from 3.83 BL/s to 6.13 BL/s are suitable for the prolonged model, while data greater than 6.13 BL/s are suitable for the sprint model. Log(scale) refers to the Weibull scale, which describes the distribution around the mean value.

Model	Variable	Coefficient	SE	*p*
Prolonged	Intercept	6.29	1.10	<0.0001
	*V_s_* (BL/s)	−0.94	0.15	<0.0001
	*BL* (m)	6.98	2.65	0.0086
	Log(scale)	−0.86	0.18	<0.0001
	*N* (prolonged)	47		
Sprint	Intercept	9.87	1.45	<0.0001
	*V_s_* (BL/s)	−0.48	0.12	<0.0001
	*BL* (m)	−7.81	2.12	0.00024
	Log(scale)	−0.50	0.10	<0.0001
	*N* (sprint)	138		

These are accelerated failure time models (Equation (5)), showing the effect of *V_s_* and *BL* on *T*. The breakpoint between prolonged models and sprint models was determined based on the optimal AIC value and C-index.

## Data Availability

Data are available from the corresponding author upon reasonable request.

## Supplementary Materials

The following supporting information can be downloaded at: https://www.mdpi.com/article/10.3390/ani15223327/s1, Figure S1: Cross-sectional velocity distribution in the flume, with data from monitoring cross section 12, grouped by nominal flow velocity: (a) 1.80 m/s; (b) 2.00 m/s; (c) 2.20 m/s; (d) 2.40 m/s; (e) 2.50 m/s; Figure S2: Correlation and collinearity tests results: (a) Correlation of hydraulic factors, (b) Collinearity test of hydraulic factors; Table S1: Approximate significance of smoothing parameters for optimal RSF-GAMMs.
