# Supplementary material for: Swimming Performance and Behavior of High-Altitude Fish in High-Flow Velocity Environments
Source: Animals (Basel). 2025 Nov 18;15(22):3327. doi: 10.3390/ani15223327 (PMC12649380; doi:10.3390/ani15223327)
Supplement: Supplementary file 1 [file animals-15-03327-s001.zip › Supplementary Materials.pdf]

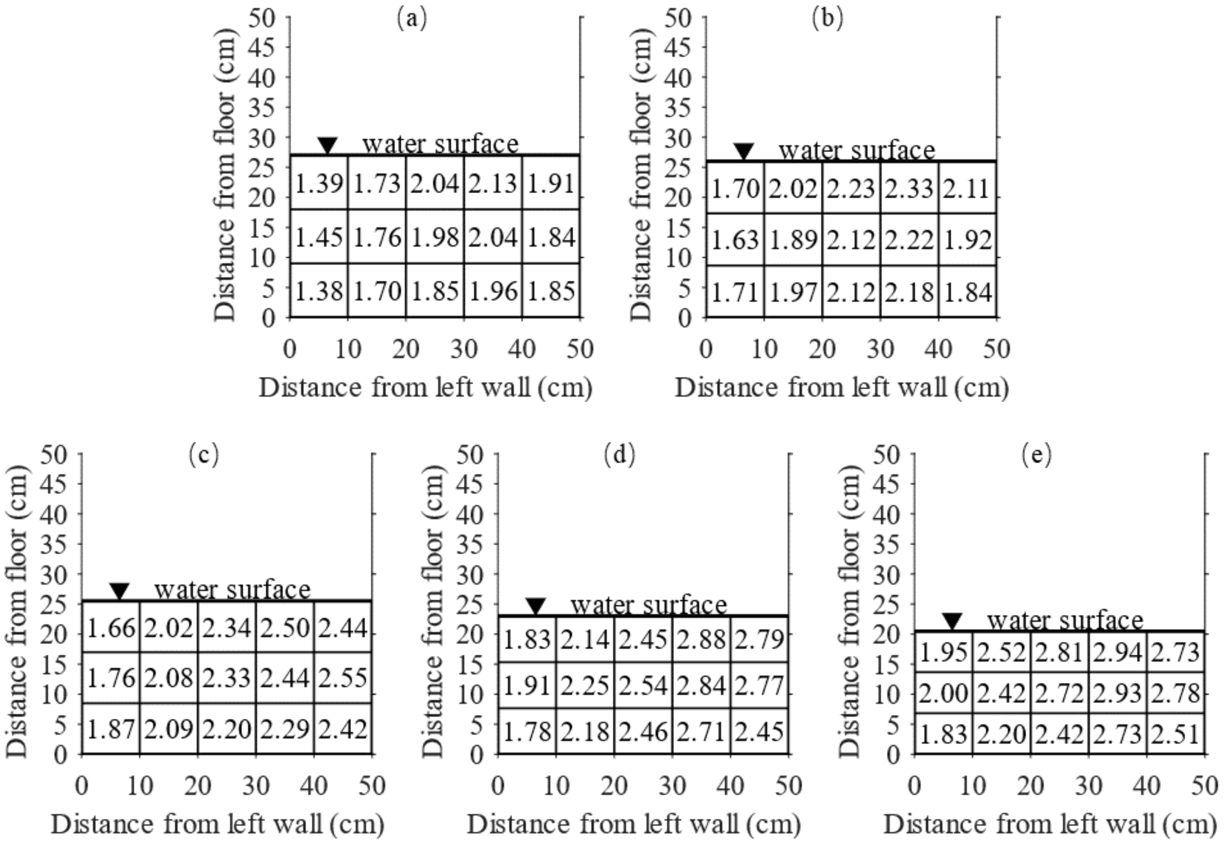

Figure S1. Cross-sectional velocity distribution in the flume, with data from monitoring cross section 12, grouped by nominal flow velocity: (a) 1.80 m/s; (b) 2.00 m/s; (c) 2.20 m/s; (d) 2.40 m/s; (e) 2.50 m/s.

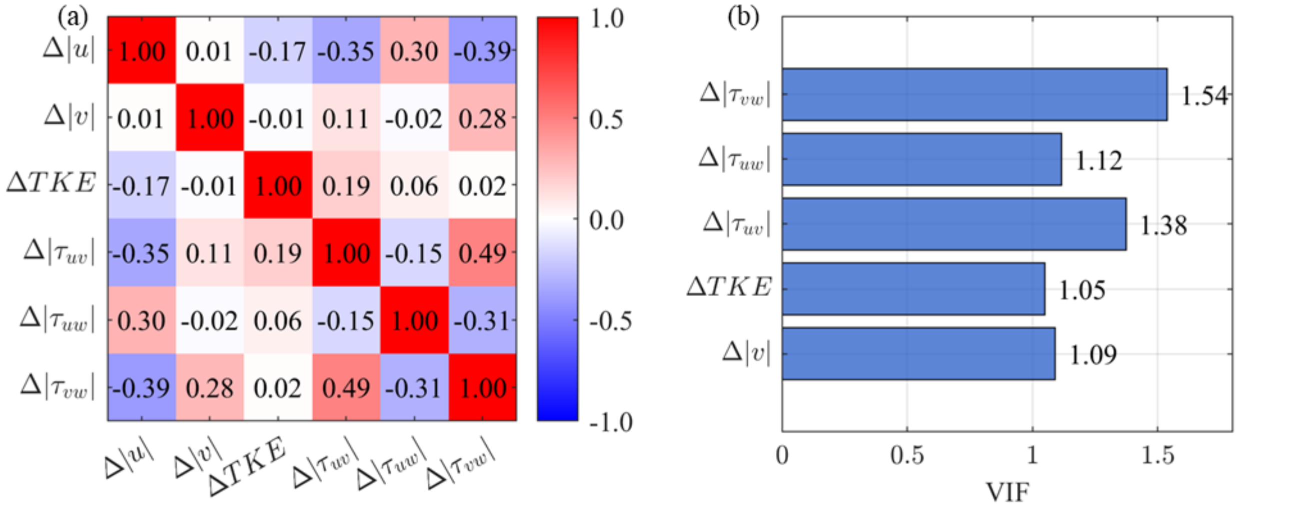

Figure S2. Correlation and collinearity tests results: (a) Correlation of hydraulic factors, (b) Collinearity test of hydraulic factors.

Table S1. Approximate significance of smoothing parameters for optimal RSF-GAMMs

Smoothing functions	edf *	Chi.sq *	p
s($\Delta\lvert u\rvert$):*Mo*-cross	0.66	2.47	0.050
s($\Delta\lvert u\rvert$):*Mo*-downstream	2.87	2398.09	0.004
s($\Delta\lvert u\rvert$):*Mo*-upstream	2.88	826.16	<0.001
s($\Delta\lvert v\rvert$):*Mo*-cross	0.87	6.95	0.028
s($\Delta\lvert v\rvert$):*Mo*-downstream	2.55	51.26	0.003
s($\Delta\lvert v\rvert$):*Mo*-upstream	0.72	3.42	0.163
s(ΔTKE):*Mo*-cross	0.88	7.46	<0.001
s(ΔTKE):*Mo*-downstream	0.28	0.54	<0.001
s(ΔTKE):*Mo*-upstream	2.79	43.73	<0.001
s($\Delta\lvert\tau_{uv}\rvert$):*Mo*-cross	2.22	15.21	<0.001
s($\Delta\lvert\tau_{uv}\rvert$):*Mo*-downstream	2.37	340.98	<0.001
s($\Delta\lvert\tau_{uv}\rvert$):*Mo*-upstream	2.81	426.54	<0.001
s($\Delta\lvert\tau_{uw}\rvert$):*Mo*-cross	1.90	64.44	<0.001
s($\Delta\lvert\tau_{uw}\rvert$):*Mo*-downstream	0.76	4.22	0.018
s($\Delta\lvert\tau_{uw}\rvert$):*Mo*-upstream	1.99	451.79	<0.001
s($\Delta\lvert\tau_{vw}\rvert$):*Mo*-cross	2.35	51.73	<0.001
s($\Delta\lvert\tau_{vw}\rvert$):*Mo*-downstream	2.87	1390.82	<0.001
s($\Delta\lvert\tau_{vw}\rvert$):*Mo*-upstream	2.98	568.14	<0.001
ti(E, $\Delta\lvert u\rvert$)	5.38	184.86	<0.001
ti(E, $\Delta\lvert v\rvert$)	2.42	8.16	0.002
ti(E, ΔTKE)	2.29	19.97	<0.001
ti(E, $\Delta\lvert\tau_{uw}\rvert$)	4.96	33.90	<0.001
ti(E, $\Delta\lvert\tau_{vw}\rvert$)	6.87	43.01	<0.001
s(*Mo*)	2.00	2654.60	<0.001
s(*fish_id*)	54.39	306.98	<0.001

* edf, Effective Degrees of Freedom; Chi.sq, Chi-square Statistic.
